# Influence of *Clear^T^* and *Clear^T2^* Agitation Conditions in the Fluorescence Imaging of 3D Spheroids

**DOI:** 10.3390/ijms22010266

**Published:** 2020-12-29

**Authors:** Daniel N. Silva, Elisabete C. Costa, Carolina F. Rodrigues, Duarte de Melo-Diogo, Ilídio J. Correia, André F. Moreira

**Affiliations:** 1 CICS-UBI—Health Sciences Research Centre, Universidade da Beira Interior, 6200-506 Covilhã, Portugal; danyelnsilva@hotmail.com (D.N.S.); elisabeteccosta@fcsaude.ubi.pt (E.C.C.); carolina.felix.rodrigues@ubi.pt (C.F.R.); demelodiogo@fcsaude.ubi.pt (D.d.M.-D.); 2 CIEPQPF—Departamento de Engenharia Química, Universidade de Coimbra, Rua Sílvio Lima, Polo II, 3030-790 Coimbra, Portugal

**Keywords:** *Clear^T^*, *Clear^T2^*, confocal microcopy, propidium iodide, tumor spheroids

## Abstract

3D tumor spheroids have arisen in the last years as potent tools for the in vitro screening of novel anticancer therapeutics. Nevertheless, to increase the reproducibility and predictability of the data originated from the spheroids it is still necessary to develop or optimize the techniques used for spheroids’ physical and biomolecular characterization. Fluorescence microscopy, such as confocal laser scanning microscopy (CLSM), is a tool commonly used by researchers to characterize spheroids structure and the antitumoral effect of novel therapeutics. However, its application in spheroids’ analysis is hindered by the limited light penetration in thick samples. For this purpose, optical clearing solutions have been explored to increase the spheroids’ transparency by reducing the light scattering. In this study, the influence of agitation conditions (i.e., static, horizontal agitation, and rotatory agitation) on the *Clear^T^* and *Clear^T2^* methods’ clearing efficacy and tumor spheroids’ imaging by CLSM was characterized. The obtained results demonstrate that the *Clear^T^* method results in the improved imaging of the spheroids interior, whereas the *Clear^T2^* resulted in an increased propidium iodide mean fluorescence intensity as well as a higher signal depth in the Z-axis. Additionally, for both methods, the best clearing results were obtained for the spheroids treated under the rotatory agitation. In general, this work provides new insights on the *Clear^T^* and *Clear^T2^* clearing methodologies and their utilization for improving the reproducibility of the data obtained through the CLSM, such as the analysis of the cell death in response to therapeutics administration.

## 1. Introduction

For the past decades, two-dimensional (2D) cultures (monolayers of cells) of tumor cells have been the gold standard to develop and evaluate the effectiveness of anticancer therapeutic agents [1]. Although these 2D cell cultures are cheap and easy to reproduce, they fail to mimic the tumor tissues’ three-dimensional (3D) architecture, their mechanic and biochemical properties, as well as their cell-cell and cell-extracellular matrix (ECM) interactions [1,2]. Up to now, these cell culture models have led to poor predictions of the therapeutic effectiveness of new therapeutics in humans [3]. To overcome this situation, 3D cell cultures such as spheroids, have emerged as an alternative for drug screening, before the study proceeds to animal experimentation or to clinical trials in humans [4]. Spheroids are able to represent several properties of the solid tumors, in comparison to 2D cell cultures, such as: (i) distribution of proliferative, senescent and necrotic cells; (ii) distribution of nutrients and gases, that leads to the formation of hypoxic and acidic environments; (iii) cell-cell signaling; (iv) ECM deposition; (v) ECM-cell and cell-cell physical interactions; (v) growth kinetics; (vi) gene expression and (vii) drug resistance (reviewed in detail in [5]). Despite being a good replica of what happens in real cancerous tissue, there is a lack of well-established protocols and equipment adapted to spheroids’ analysis hindering the reproducibility and accuracy of the data originated from these models, i.e., most of the currently available methods are only optimized for the analysis of the therapeutics effectiveness on 2D cell cultures [2,6,7]. For instance, the imaging of spheroids by fluorescence microscopy is quite challenging, namely through confocal laser scanning microscopy (CLSM). This technique, which is one of the most common and widely available in the different laboratories worldwide, is used to analyze several features of the spheroids (e.g., size and morphology [8,9], proteins expression [10], cellular death [11], and therapeutics or nanoparticles penetration [12]). Nevertheless, since CLSM does not allow one to fully image samples with a thickness higher than 100–150 μm [13,14,15], it is impossible to analyze whole intact spheroids, which usually present diameters superior to 400 µm [16]. Although this problem can be overcome by sectioning the spheroid into thin slices of 5–7 µm, this procedure is very time-consuming, depend on organic solvent, and can also compromise the initial structure of the spheroid [13,17]. Therefore, optical clearing methods have been evaluated to improve the imaging of spheroids by CLSM [18,19,20,21,22]. These methods aim to improve the microscope’s signal penetration depth by increasing the spheroids transparency and improve the light penetration within the spheroid. For that purpose, the clearing solutions promote the homogenization of the refractive index (RI) through the spheroids and reduce the light scattering effect [15,23]. In the literature, several optical clearing methods have been used for improving the spheroids analysis, namely *Clear^T^* [19], *Clear^T2^* [18,20,21], SeeDB [14,18], FocusClear^™^ [22], BABB [24,25,26,27], Scale [14,18], CUBIC [28], and CLARITY [29,30]. Among these, *Clear^T^* and *Clear^T2^* methods are organic-solvent and detergent free, the solutions are easy to handle, and the protocols are not very time-consuming. In *Clear^T^* method, samples are cleared by immersing them in increasing concentrations of aqueous solutions of formamide. The *Clear^T2^* method is performed by immersing the samples in aqueous solutions with increasing concentrations of formamide and polyethylene glycol (PEG). PEG is used to maintain the integrity and stability of the fluorescently-labeled elements (e.g., proteins), thus avoiding the fluorescence quenching prompted by the formamide [15,31,32,33]. Both protocols were described for the first time by Kuwajima et al. to clear mice tissues (e.g., whole brain and brain sections) [32].

In our group, we already demonstrated that *Clear^T^* and *Clear^T2^* enhance the imaging of propidium iodide (PI)-stained spheroids via CLSM [19,21]. However, the influence of the *Clear^T^* and *Clear^T2^* clearing solutions incubation conditions (e.g., static, horizontal agitation and rotatory agitation) on the spheroids clearing and imaging by CLSM is still poorly explored in the literature. In most of the studies, the spheroids are maintained under horizontal agitation during their incubation with the clearing solutions [18,21]. For instance, Boutin et al. performed the *Clear^T2^* method using a shaker plate at 90 rotations per minute (RPM) [18]. In other studies, it is not described as the conditions used for the incubation of the *Clear^T^* and *Clear^T2^* [32,34]. In this work, a direct comparison of the *Clear^T^* and *Clear^T2^* methods clearing efficacy was performed taking also in account the influence of the solutions incubation conditions (static, horizontal agitation, and rotatory agitation) on the spheroids’ transparency and imaging by CLSM, to determine the best procedure to obtain improved images of PI-stained spheroids.

## 2. Materials and Methods

### 2.1. Materials

Normal human dermal fibroblasts (HFIB) were acquired from PromoCell (Labclinics, S.A., Barcelona, Spain). T-flasks, cell culture plates and other cell culture consumables were obtained from ThermoFisher Scientific (Porto, Portugal). Cell imaging plates were bought from Ibidi GmbH (Ibidi, Munich, Germany). Fetal bovine serum (FBS) was purchased from Biochrom AG (Berlin, Germany). PI was procured from Invitrogen (Carlsbad, CA, USA). Agarose was obtained from Grisp (Porto, Portugal). Dulbecco’s modified Eagle’s medium F12 (DMEM-F12), paraformaldehyde (PFA), phosphate-buffered saline (PBS), trypsin, formamide (≥99.5%), ethylenediaminetetraacetate (EDTA), and PEG 4000 Da were purchased from Sigma-Aldrich (Sintra, Portugal).

### 2.2. Methods

#### 2.2.1. Cells Maintenance and 3D HFIB Formation

HFIB cells were cultured in 175 cm^2^ T-flasks with DMEM-F12 media supplemented with FBS (10% (*v*/*v*)) and gentamycin and streptomycin (1% (*v*/*v*)), inside an incubator with a humidified atmosphere at 37 °C and 5 % CO_2_ [35]. When cells achieved a confluent state, they were recovered by using 0.25 % trypsin (1:250) and EDTA 0.1% (*w*/*v*). The spheroids self-assembly on agarose structures with 81 spherical microwells was performed as previously described in our group [19,21]. In brief, an agarose solution (2% (*w*/*v*) in H_2_O) was placed on 3-D Petri Dish^®^ templates (Microtissues Inc., Providence, RI, USA) to obtain the agarose structures. After, these structures were placed in 12-well cell culture plates and sterilized by ultraviolet (UV) radiation. Then, 1 × 10^6^ HFIB cells were placed in each structure. During the spheroids’ growth, DMEM-F12 media containing 10% (*v*/*v*) of FBS and 1% (*v*/*v*) of streptomycin and gentamycin was added to each well and the plates were maintained inside an incubator with a humidified atmosphere (37 °C and 5% CO_2_). The medium was changed every two days.

#### 2.2.2. HFIB Spheroids Fixation and Incubation with PI

After 6 days, spheroids were collected and subjected to a chemical fixation procedure. In brief, spheroids were immersed on 4% (*w*/*v*) PFA during 24 h at 4 °C [21]. After, spheroids were washed with PBS and labeled with PI (1 mL; 10 μg/mL in H_2_O). After 24 h of PI incubation at 4 °C, spheroids were washed three times with PBS to remove the excess of PI staining.

#### 2.2.3. Spheroids Optical Clearing by Clear^T^

**Figure 1:**
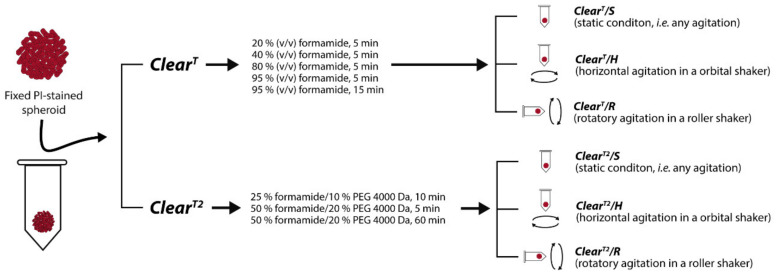
Overview of the PI-stained spheroids optical clearing performed through *Clear^T^* and *Clear^T2^* using different spheroids immersion conditions, namely static, horizontal agitation, and rotatory agitation.

Spheroids that were previously stained with PI were cleared by using the *Clear^T^* solutions [19,32] (Figure 1). Briefly, different formamide solutions (20, 40, 80 and 95% (*v*/*v*) in PBS) were freshly prepared. Then, spheroids were serially incubated with 20, 40, 80 and 95% formamide solutions during 5 min each, and lastly with 95% formamide solution during 15 min. To assess the best conditions for spheroids’ clearing, spheroids were immersed in the clearing solutions under three different conditions: (i) static conditions where no agitation was used—*Clear^T^/S*; (ii) horizontal agitation at 200 RPM by using an orbital mixer (LBX MM1500 series, Labbox, Barcelona, Spain)—*Clear^T^/H*; and (iii) rotatory agitation using a tube roller shaker (MX-T6-S Analog Tube Roller, Scilogex, city, CT, USA) at 50 RPM—*Clear^T^/R*. All the methods were performed at room temperature (RT). After the clearing, spheroids were transferred to μ-slide 8 well imaging plates (Ibidi GmbH) to be imaged by CLSM (Section 2.2.6).

#### 2.2.4. Spheroids Optical Clearing by *Clear^T2^*

PI-stained spheroids were cleared by using *Clear^T2^* solutions [21,32]—Figure 1. Initially, 25% formamide/10% PEG 4000 Da and 50% formamide/20% PEG 4000 Da solutions were freshly prepared, as previously described in detail by Kuwajima [32]. Then, spheroids were incubated with 25% formamide/10% PEG solution during 10 min, 50% formamide/20% PEG for 5 min and lastly 50% formamide/20% PEG for 1 h. During the clearing procedure, three different conditions were tested, as described in Section 2.2.3 for the *Clear^T^* method: (i) *Clear^T2^/S*; (ii) *Clear^T2^/H*; and (iii) *Clear^T2^/R*. All methods were performed at RT. After the clearing, spheroids were transferred to μ-slide 8 well imaging plates (Ibidi GmbH) to be imaged via CLSM (Section 2.2.6).

#### 2.2.5. Spheroids Imaging and Analysis by Optical Microscopy

To observe any possible variation in HFIB spheroids transparency and size after each optical clearing protocol is performed, optical microscopy images of the spheroids were acquired using a CX41 inverted optical microscope (Olympus, Hamburg, Germany) equipped with an Olympus SP-500 UZ digital camera and an Axio Imager A1 inverted microscope (Carl Zeiss, SMT, Inc., Oberkochen, Germany) equipped with an AxioVision camera. The transparency was checked by placing the spheroids on a crosshatched background, as previously described elsewhere [18,19,21,32]. The influence of the optical clearing methods on the size of the spheroids was determined by measuring their diameter before and after the clearing procedure. For this analysis, the optical microscopy images were analyzed by using an image processing program designed for scientific purposes—ImageJ (National Institutes of Health, Bethesda, MD, USA) [36], as previously described in detail [35].

#### 2.2.6. Spheroids Imaging and Analysis by CLSM

HFIB spheroids were imaged by CLSM using a Zeiss LSM 710 AxioObserver laser scanning confocal microscope. For comparative purposes, all the samples were analyzed using the same equipment settings. The objective used was a 10× air objective (EC Plan-Neofluar 10×/0.30 M27). The size of the confocal aperture was 1 Airy disk, and z-stacks were collected with 5 μm intervals. Laser power and master gain were kept constant during image acquisition. PI was visualized by using 514 nm argon laser and the emission wavelengths range was 566–719 nm.

The images obtained through CLSM were then analyzed by using ImageJ (National Institutes of Health [36]), to determine the PI fluorescence levels, imaging depth of the PI in the Z-axis and PI cross-section imaging depth. The PI fluorescence levels were obtained by calculating the corrected total cell fluorescence (CTCF) [14,37,38]. For this purpose, the integrated density (ID), the spheroids area (A) and mean fluorescence of the background (MFB) values of the CLSM maximum intensity z-projections with a thickness of 100 µm (total of 20 Z-stacks) were determined and then used in the Equation (1) [19,21,37]:

(1) CTCF=ID−A × MFB

The imaging penetration depth of the PI, i.e., how deep in the Z-axis it is possible to acquire PI fluorescence signal (please see Figure 4A), was calculated by multiplying the number of stacks with PI fluorescence (stacks with fluorescence intensity in the spheroids area higher than the fluorescence intensity of the background) by the thickness of the stack (5 µm) [39,40]. Lastly, to determine the PI cross-section imaging depth that translates the PI fluorescence inside the spheroid, it was counted the number of pixels with fluorescence in the spheroid area at 25, 50, 75 and 100 µm of depth in the Z-axis (please see Figure 4D) [41,42]. To accomplish that, the gray value of each pixel in the spheroid area was determined (gray value equal to zero corresponds to pixels with no PI fluorescence; gray value higher than 0 corresponds to pixels with PI fluorescence) [41,42].

The obtained values were compared and normalized to the average of those obtained for non-cleared spheroids, i.e., maintained in PBS.

#### 2.2.7. Statistical Analysis

The data obtained were expressed as mean value ± standard deviation (S.D.). The statistical analysis was performed using one-way analysis of variance (ANOVA) test. A value of *p* < 0.05 was considered statistically significant. Data analysis was performed in GraphPad Prism v.6.0 software (trial version, GraphPad Software, San Diego, CA, USA).

## 3. Results and Discussion

The optical clearing methods have allowed the obtention of fluorescence microscopy images of 3D cell cultures with better quality and resolution. Nevertheless, these methods were initially developed to be applied in tissues or organs gathered from animals and very few have been optimized for the imaging of spheroids. *Clear^T^* and *Clear^T2^* methods have been already used to clear spheroids in several works [18,19,20,21], although these methods are not yet fully optimized for this purpose. In a previous study performed by our group, we investigated the influence of PEG molecular weight (4000, 8000 and 10,000 Da) on the spheroids clearing efficacy of the *Clear^T2^* method [21]. Such study demonstrated that the utilization of PEG 4000 Da, instead of the conventional PEG 8000 Da, allows better imaging of spheroids, since it was possible to obtain higher PI mean fluorescence intensity as well as an increased signal penetration in the Z-axis and fluorescence signal in the interior of the spheroids [21]. Herein, we further optimized both *Clear^T^* and *Clear^T2^* methods taking into consideration the conditions of incubation of the clearing solutions (Figure 1). For that purpose, PI-stained HFIB spheroids were cleared by using aqueous solutions of formamide (*Clear^T^*) and formamide:PEG 4000 Da (*Clear^T2^*) and incubation under different conditions, namely: (i) static; (ii) horizontal agitation using an orbital mixer; and (iii) rotatory agitation using a roller shaker. After the clearing and imaging by CLSM, the spheroids’ size, transparency, PI fluorescence intensity, PI imaging depth in the Z-axis and PI cross-section imaging were analyzed to determine the best approach to perform the *Clear^T^* and *Clear^T2^* methods.

### 3.1. Spheroids’ Size

**Figure 2:**
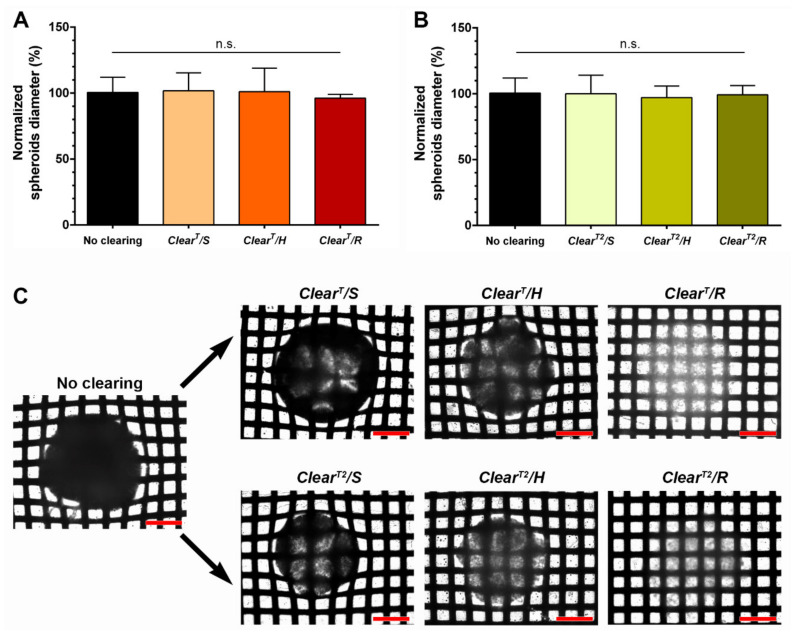
Influence of the immersion conditions used to perform the *Clear^T^* (**A**) and *Clear^T2^* (**B**) methods on HFIB spheroids size and transparency. (**A**,**B**) represent the spheroids’ diameter after they were cleared with *Clear^T^* and *Clear^T2^* methods under static, horizontal and rotatory agitation, normalized to the average diameter of non-cleared spheroids (388.97 ± 42.94 µm). Optical transparency of a non-cleared spheroid and spheroids cleared by using *Clear^T^* and *Clear^T2^* methods under static, horizontal agitation, and rotatory agitation conditions (**C**). Spheroids were imaged on crosshatched backgrounds for the relative differences in transparency be noticeable. Scale bars correspond to 200 μm. Data are presented as mean ± S.D.; *n* = 7, *p* < 0.05, n.s.—non-significant.

The variations on spheroids’ size are one of the major concerns associated with the use of the optical clearing methods since it means that the methods affect the sample initial structure and morphology [15,38]. In some optical clearing methods, organic solvents like methanol are used for promoting samples dehydration (e.g., Spalteholz’s technique, BABB and 3DISCO), and these solvents induce spheroids shrinkage [43]. Likewise, methods that include hydration steps using urea (e.g., Sca*l*e and CUBIC) usually induce spheroids swelling [31]. Theoretically, *Clear^T^* and *Clear^T2^* methods may also increase the volume of the samples, since these methods include the intake of an aqueous clearing solution into the sample by osmotic pressure [15]. Nevertheless, in previous studies, it was demonstrated that *Clear^T^* and *Clear^T2^* methods do not induce significative changes in the size of animal samples (e.g., mouse brains) [32] or spheroids [18,19,21]. Similarly, in this study, it was also observed that the spheroids’ size is not affected by the incubation conditions of the solutions used in the *Clear^T^* and *Clear^T2^* methods (Figure 2 and Table 1). Before the clearing process, spheroids exhibited mean diameters of 399.04 ± 39.26 µm. After the performance of the *Clear^T^* method under static, horizontal agitation and rotatory agitation conditions, the mean diameter of the spheroids was 473.23 ± 63.24, 469.90 ± 83.18 and 447.21 ± 13.71 µm, respectively (Figure 2A). Similar results were obtained for the *Clear^T2^* method, i.e., the spheroids’ mean diameter was 478.91 ± 67.10, 464.77 ± 42.24 and 474.91 ± 33.43 µm after using static, horizontal agitation or rotatory agitation conditions, respectively (Figure 2B). These results demonstrate that the agitation conditions used for the incubation of the formamide-based solutions will not influence spheroids structure, and therefore it will not lead to incorrect interpretations [15,38].

### 3.2. Spheroids’ Transparency

Optical microscopy images of the spheroids placed on a crosshatched background were acquired to observe if the agitation conditions affect the *Clear^T^* and *Clear^T2^* methods capacity to render transparency to the spheroids [18,19,21,32]. As it is possible to observe in Figure 2C, independently of the incubation condition of the different formamide-based solutions, all the cleared spheroids become more transparent when compared to the non-cleared ones. Notwithstanding, it is possible to witness that the utilization of rotatory agitation during the spheroids immersion in the clearing solutions, i.e., *Clear^T^/R* and *Clear^T2^/R* methods, resulted in the greatest spheroids transparency (Figure 2C).

### 3.3. PI Fluorescence

It is important to ensure that the optical clearing methods do not impact the fluorescence intensity, e.g., quenching. Preservation of the fluorescence will allow the imaging of the sample through fluorescence microscopy and the correct analysis of the labeled proteins and cellular structures [19]. Methods that include dehydration steps (e.g., BABB and 3DISCO) usually alter the fluorescence of proteins, since protein-based fluorescent chromophores need water molecules to sustain their fluorescence emission [15]. Additionally, these methods use organic solvents to clear the samples that can induce structural changes on the fluorescence labelled proteins disrupting their fluorescence [15,44]. Otherwise, it is also known that the formamide used in *Clear^T^* and *Clear^T2^* can mediate the denaturation of proteins leading to the fluorescence quenching, as previously demonstrated using in vivo samples stained with the green fluorescent protein (GFP) [32]. With this in mind, *Clear^T2^* method uses PEG simultaneously with formamide to stabilize the proteins, avoiding the formamide-mediated quenching of the protein dyes fluorescence [32,38].

**Figure 3:**
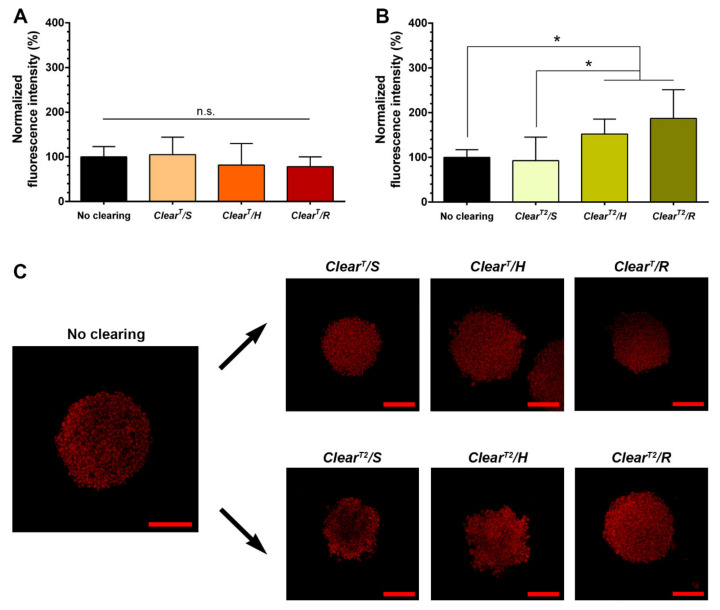
Influence of the immersion conditions used to perform the *Clear^T^* and *Clear^T2^* on the PI fluorescence intensity in HFIB spheroids. Fluorescence intensity of PI-stained spheroids after clearing with *Clear^T^* (**A**) and *Clear^T2^* (**B**) methods under static, horizontal agitation, and rotatory agitation, normalized to the average of the non-cleared spheroids fluorescence intensity. Maximum intensity projections CLSM images with a thickness of 100 µm of non-cleared spheroids and spheroids cleared by *Clear^T^* and *Clear^T2^* methods under static, horizontal agitation, and rotatory agitation (**C**). Scale bars correspond to 200 µm; *n* = 9, * *p* < 0.05, n.s.—non-significant.

Considering that the incubation conditions of the *Clear^T^* and *Clear^T2^* clearing solutions resulted in different levels of spheroids transparency, it was also evaluated if the PI fluorescence intensity is influenced by the samples agitation. Therefore, the PI fluorescence intensity of maximum projections (100 µm) CLSM images of non-cleared and spheroids cleared by *Clear^T^* and *Clear^T2^* under different conditions were measured and are presented in Figure 3. The obtained images show that the *Clear^T^/S* and *Clear^T^/H* did not lead to the loss of PI-fluorescence, while the *Clear^T^/R* method reduced the PI fluorescence significantly to 59.26 ± 10.39% (Figure 3A,C). Such results can be attributed to the DNA denaturation [45] and the quenching of fluorescent dyes [46] by the formamide. Therefore, the reduction on the PI fluorescence on *Clear^T^/R* may show that the rotatory agitation increases the amount of formamide that interact with the spheroids when compared to the static and horizontal agitation conditions. Otherwise, the spheroids cleared using the *Clear^T2^/H* and *Clear^T2^/R* methods showed a higher PI fluorescence intensity, 152.11 ± 33.48% and 187 ± 64.25%, respectively (Figure 3B,C). Such can be attributed to the increased interaction of *Clear^T2^* solutions with the spheroids under agitation conditions, favoring the stabilization of the PI/DNA staining by PEG as previously reported in the literature [21]. Furthermore, considering that formamide:PEG solutions do not have absorbance in 400–800 nm range, possible autofluorescence phenomena of these solutions should be disregarded, as previously showed elsewhere [21,47,48].

### 3.4. PI Fluorescence Depth in Z-Axis

**Figure 4:**
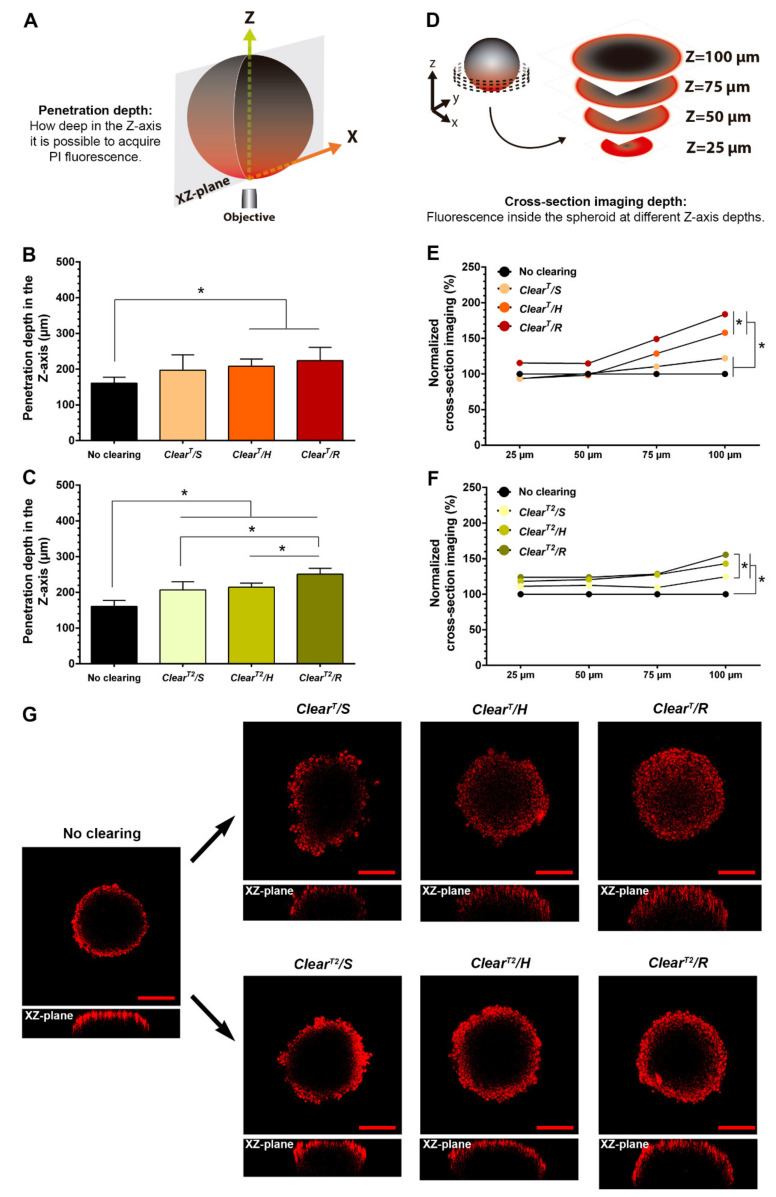
Influence of the immersion conditions used to perform the *Clear^T^* and *Clear^T2^* methods on the PI imaging depth in the Z-axis and the PI cross-section imaging depth in HFIB spheroids. Schematic representation of the penetration depth in the Z-axis measured in the spheroids (**A**). The penetration depth of PI fluorescence signal in the Z-axis of non-cleared spheroids and spheroids cleared by using *Clear^T^* (**B**) and *Clear^T2^* (**C**) methods under static, horizontal agitation, and rotatory agitation conditions. Schematic representation of the PI cross-section imaging depth measured in the spheroids (**D**). Cross-section imaging depth of PI fluorescence signal of spheroids cleared by using *Clear^T^* (**E**) and *Clear^T2^* (**F**) methods under static, horizontal agitation, and rotatory agitation conditions, normalized to the average of the non-cleared spheroids values. Cross-section CLSM images of non-cleared and cleared spheroids by using *Clear^T^* and *Clear^T2^* methods under static, horizontal and rotatory agitation conditions (**G**). Scale bars correspond to 200 µm; *n* = 9, * *p* < 0.05, n.s.—non-significant.

Whole thick samples (like spheroids) are difficult to be fully imaged by CLSM since the fluorescence signal depth in the Z-axis is very limited. To surpass this limitation, both *Clear^T^* and *Clear^T2^* methods have demonstrated to be capable of increasing the fluorescence imaging depth in the Z-axis on spheroids [18,19,20,21]. The clearing solutions of these methods homogenize the spheroids overall RI to about 1.44, which leads to a reduction of the light dispersion and improves the excitation and emission light penetration [15]. The better RI homogenization, the greater will be the spheroids transparency and then the traveling of the light throughout the spheroids [33]. Since the transparency of the spheroids is depended on the procedure used to immerse them on the clearing solutions, this may also influence the imaging depth of the PI in the Z-axis. To evaluate such effect, the PI signal depth in the Z-axis was characterized in non-cleared spheroids and those cleared by *Clear^T^/S, Clear^T^/H*, *Clear^T^/R, Clear^T2^/S*, *Clear^T2^/H and Clear^T2^/R* (Figure 4A–C).

The attained results showed that both optical clearing methods allowed to acquire fluorescence at deeper distances in the Z-axis (Figure 4B,C). Such effect was more pronounced when the spheroids were immersed in the clearing solutions under horizontal and/or rotatory agitation. In comparison to the non-cleared spheroids, it was possible to obtain more 29.84 ± 12.41% and 39.53 ± 23.31% PI signal in the Z-axis after the spheroids being cleared by *Clear^T^/H* and C*lear^T^/R*, respectively (Figure 4B), and more 33.60 ± 7.15% and 56.60 ± 10.40% when the *Clear^T2^/H* and *Clear^T2^/R* methods were used, respectively (Figure 4C). These results are in agreement with those previously obtained and demonstrate that the use of agitation improves the penetration of the clearing solutions into the spheroids, allowing a better RI homogenization and then a higher spheroids transparency and detection of the fluorescence signal at higher depths in the Z-axis.

### 3.5. Spheroids Cross-Section Imaging

The acquisition of fluorescence inside the spheroids is very important for therapeutics research purposes. For instance, the imaging of the interior of the spheroids with a fluorescence microscope is crucial to evaluate the dispersion of pharmaceuticals or nanoparticles within the spheroids [49,50,51], as well as to analyze the cellular death and the expression of proteins before and after therapeutics administration [52,53,54]. Nevertheless, the light scattering and its dispersion through the sample hinder the imaging of the spheroids’ interior [18]. Therefore, the cross-section imaging depth in the PI-stained spheroids subjected to *Clear^T^* and *Clear^T2^* methods was performed to determine the best incubation conditions. For that purpose, the number of pixels with PI signal inside the spheroids at different depths in Z-axis (25, 50, 75 and 100 µm) was measured [41,42]—Figure 4D.

The obtained results showed that both *Clear^T^* and *Clear^T2^* methods allowed to obtain a higher PI fluorescence inside the spheroids when compared to the non-cleared spheroids (Figure 4E–G), being this difference noticeable at 100 µm in the Z-axis. Moreover, the use of agitation during the spheroids incubation with the clearing solutions was one more time crucial to improve the imaging of the interior of the spheroids. In fact, at 100 µm in the Z-axis, the *Clear^T^/H* and *Clear^T2^/H* methods allowed to obtain more 57.80 ± 18.17 % and 43.00 ± 15.32 % of PI signal inside the spheroids, whereas the *Clear^T^/R* and *Clear^T2^/R* enhanced the PI signal in 83.79 ± 40.14% and 55.43 ± 31.74%, respectively (Figure 4E,F). Moreover, the CLSM images presented in Figure 4G further support these results, being observed PI-stained cells within deeper regions of the spheroids at a penetration depth of 100 µm when horizontal or rotatory agitation conditions were used, which contrasts with more localize PI fluorescence in the most external layers of the non-cleared or cleared by *Clear^T^/S* and *Clear^T2^/S* spheroids. Interestingly, the *Clear^T^* method allowed to obtain more PI signal inside the spheroids in comparison to those cleared through the *Clear^T2^* method (Figure 4E–G). Such result may be explained by an easier entrance of the formamide-aqueous solutions into the spheroids’ interior, since these are less viscous than the formamide/PEG solutions used in the *Clear^T2^* method.

## 4. Conclusions

The potential of spheroid tumor models for the in vitro screening of novel anticancer therapeutic approaches was already demonstrated in the literature. Nevertheless, the methods and equipment for extracting data from these models still need to be optimized for application in spheroids. In this field, different studies have been demonstrating that optical clearing methods are crucial for the imaging and analysis of whole spheroids, avoiding the sample sectioning. In this work, the clearing efficacy of *Clear^T^* and *Clear^T2^* optical clearing methods and the influence of the incubation conditions of their solutions (static, horizontal agitation, and rotatory agitation) have on the imaging of PI-stained spheroids by CLSM were analyzed (Table 1). Both *Clear^T^* and *Clear^T2^* methods can be used to prompt transparency on the spheroids, without affecting their size. *Clear^T^* demonstrated to be the best method to image the spheroids’ interior, while *Clear^T2^* allowed to achieve a greater PI fluorescence intensity and a higher PI signal depth in the Z-axis. In both methods, the use of agitation during the spheroids immersion in the clearing solutions resulted in improved imaging of the PI fluorescence in the spheroids, principally when rotatory agitation was employed.

Overall, this article contributes to increasing the amount and accuracy of the data extracted from spheroid models. Additionally, the obtained data can assist other researchers to further optimize the optical clearing methods that will enhance the imaging of whole spheroids. Such is crucial for supporting the application of these models in pharmaceutical research and industry, for instance, to monitor by fluorescence microscopy the cellular death in spheroids prompted by novel anticancer therapeutics.

## Figures and Tables

**Table 1:** Overview of *Clear^T^* and *Clear^T2^* application in PI-stained spheroids by using different incubation conditions of the clearing solutions.

Spheroid’s Analysis	No Clearing	*Clear^T^*			*Clear^T2^*		
		Static	Horizontal Agitation	Rotatory Agitation	Static	Horizontal Agitation	Rotatory Agitation
Spheroids’ size (µm)	399.04 ± 39.26	473.23 ± 63.24	469.90 ± 83.18	447.21 ± 13.71	478.91 ± 67.10	464.77 ± 42.24	474.91 ± 33.43
Spheroids’ transparency	None	Moderate	High	Very high	Moderate	High	Very High
PI fluorescence (%) ^a^	100 ± 22.94	104.86 ± 39.14	81.71 ± 47.79	59.26 ± 10.38	92.,65 ± 52.56	152.12 ± 33.48	186.93 ± 64.25
PI imaging depth in the Z-axis (µm)	160.45 ± 16.80	197.14 ± 42.71	208.33 ± 19.92	223.89 ± 37.40	206.82 ± 23.05	214.38 ± 11.48	250.77 ± 16.69
PI cross-imaging depth at 100 µm in the Z-axis (%) ^a^	100.00 ± 10.66	122.12 ± 13.48	157.80 ± 18.17	183.79 ± 40.14	124.58 ± 26.86	143.00 ± 15.32	155.43 ± 31.74

^a^: Values normalized to the average of the values obtained for non-cleared spheroids (maintained in PBS).

## Data Availability

The data presented in this study are available on request from the corresponding author. The data are not publicly available due to privacy restrictions.

## References

[1] Kapałczyńska M., Kolenda T., Przybyła W., Zajączkowska M., Teresiak A., Filas V., Ibbs M., Bliźniak R., Łuczewski Ł., Lamperska K. (2018). 2D and 3D cell cultures—A comparison of different types of cancer cell cultures. Arch. Med. Sci..

[2] Duval K., Grover H., Han L.H., Mou Y., Pegoraro A.F., Fredberg J., Chen Z. (2017). Modeling physiological events in 2D vs. 3D cell culture. Physiology.

[3] Fang Y., Eglen R.M. (2017). Three-Dimensional cell cultures in drug discovery and development. SLAS Discov..

[4] Breslin S., O’Driscoll L. (2013). Three-Dimensional cell culture: The missing link in drug discovery. Drug Discov. Today.

[5] Nunes A.S., Barros A.S., Costa E.C., Moreira A.F., Correia I.J. (2019). 3D tumor spheroids as in vitro models to mimic in vivo human solid tumors resistance to therapeutic drugs. Biotechnol. Bioeng..

[6] Van den Brand D., Massuger L.F., Brock R. (2017). Mimicking tumors: Toward more predictive in vitro models for peptide- and protein-conjugated drugs. Bioconjugate Chem..

[7] Nunes A.S., Costa E.C., Barros A.S., de Melo-Diogo D., Correia I.J. (2018). Establishment of 2D cell cultures derived from 3D MCF-7 spheroids displaying a doxorubicin resistant profile. Biotechnol. J..

[8] Gong X., Lin C., Cheng J., Su J., Zhao H., Liu T., Wen X., Zhao P. (2015). Generation of multicellular tumor spheroids with microwell-based agarose scaffolds for drug testing. PLoS ONE.

[9] Sirenko O., Mitlo T., Hesley J., Luke S., Owens W., Cromwell E.F. (2015). High-Content assays for characterizing the viability and morphology of 3D cancer spheroid cultures. Assay Drug Dev. Technol..

[10] Oltolina F., Zamperone A., Colangelo D., Gregoletto L., Reano S., Pietronave S., Merlin S., Talmon M., Novelli E., Diena M. (2015). Human cardiac progenitor spheroids exhibit enhanced engraftment potential. PLoS ONE.

[11] Neto A.I., Correia C.R., Oliveira M.B., Rial-Hermida M.I., Alvarez-Lorenzo C., Reis R.L., Mano J.F. (2015). A novel hanging spherical drop system for the generation of cellular spheroids and high throughput combinatorial drug screening. Biomater. Sci..

[12] Lu H., Utama R.H., Kitiyotsawat U., Babiuch K., Jiang Y., Stenzel M.H. (2015). Enhanced transcellular penetration and drug delivery by crosslinked polymeric micelles into pancreatic multicellular tumor spheroids. Biomater. Sci..

[13] Graf B.W., Boppart S.A. (2010). Imaging and analysis of three-dimensional cell culture models. Methods Mol. Biol..

[14] Grist S.M., Nasseri S.S., Poon T., Roskelley C., Cheung K.C. (2016). On-Chip clearing of arrays of 3-D cell cultures and micro-tissues. Biomicrofluidics.

[15] Richardson D.S., Lichtman J.W. (2015). Clarifying tissue clearing. Cell.

[16] Kunz-Schughart L.A., Freyer J.P., Hofstaedter F., Ebner R. (2004). The use of 3-D cultures for high-throughput screening: The multicellular spheroid model. J. Biomol. Screen..

[17] Costa E.C., Moreira A.F., de Melo-Diogo D., Gaspar V.M., Carvalho M.P., Correia I.J. (2016). 3D tumor spheroids: An overview on the tools and techniques used for their analysis. Biotechnol. Adv..

[18] Boutin M.E., Hoffman-Kim D. (2015). Application and assessment of optical clearing methods for imaging of tissue-engineered neural stem cell spheres. Tissue Eng..

[19] Costa E.C., Moreira A.F., de Melo-Diogo D., Correia I.J. (2018). ClearT immersion optical clearing method for intact 3D spheroids imaging through confocal laser scanning microscopy. Opt. Laser Technol..

[20] Kabadi P.K., Vantangoli M.M., Rodd A.L., Leary E., Madnick S.J., Morgan J.R., Kane A., Boekelheide K. (2015). Into the depths: Techniques for in vitro three-dimensional microtissue visualization. Biotechniques.

[21] Costa E.C., Moreira A.F., de Melo-Diogo D., Correia I.J. (2018). Polyethylene glycol molecular weight influences the Clear T2 optical clearing method for spheroids imaging by confocal laser scanning microscopy. J. Biomed. Opt..

[22] Chen Y., Tsai Y.-H., Liu Y.-A., Lee S.-H., Tseng S.-H., Tang S.-C. (2013). Application of three-dimensional imaging to the intestinal crypt organoids and biopsied intestinal tissues. Sci. World J..

[23] Yu T., Qi Y., Gong H., Luo Q., Zhu D. (2018). Optical clearing for multiscale biological tissues. J. Biophotonics.

[24] Wenzel C., Riefke B., Gründemann S., Krebs A., Christian S., Prinz F., Osterland M., Golfier S., Räse S., Ansari N. (2014). 3D high-content screening for the identification of compounds that target cells in dormant tumor spheroid regions. Exp. Cell Res..

[25] Desmaison A., Guillaume L., Triclin S., Weiss P., Ducommun B., Lobjois V. (2018). Impact of physical confinement on nuclei geometry and cell division dynamics in 3D spheroids. Sci. Rep..

[26] Schmitz A., Fischer S.C., Mattheyer C., Pampaloni F., Stelzer E.H.K. (2017). Multiscale image analysis reveals structural heterogeneity of the cell microenvironment in homotypic spheroids. Sci. Rep..

[27] Smyrek I., Stelzer E.H.K. (2017). Quantitative three-dimensional evaluation of immunofluorescence staining for large whole mount spheroids with light sheet microscopy. Biomed. Opt. Express.

[28] Masson A., Escande P., Frongia C., Clouvel G., Ducommun B., Lorenzo C. (2015). High-Resolution in-depth imaging of optically cleared thick samples using an adaptive SPIM. Sci. Rep..

[29] Santisteban T.S., Rabajania O., Kalinina I., Robinson S., Meier M. (2018). Rapid spheroid clearing on a microfluidic chip. Lab Chip.

[30] Chen Y.Y., Silva P.N., Syed A.M., Sindhwani S., Rocheleau J.V., Chan W.C. (2016). Clarifying intact 3D tissues on a microfluidic chip for high-throughput structural analysis. Proc. Natl. Acad. Sci. USA.

[31] Seo J., Choe M., Kim S.Y. (2016). Clearing and labeling techniques for large-scale biological tissues. Mol. Cells.

[32] Kuwajima T., Sitko A.A., Bhansali P., Jurgens C., Guido W., Mason C. (2013). ClearT: A detergent- and solvent-free clearing method for neuronal and non-neuronal tissue. Development.

[33] Feuchtinger A., Walch A., Dobosz M. (2016). Deep tissue imaging: A review from a preclinical cancer research perspective. Histochem. Cell Biol..

[34] Berke I.M., Miola J.P., David M.A., Smith M.K., Price C. (2016). Seeing through musculoskeletal tissues: Improving in situ imaging of bone and the lacunar canalicular system through optical clearing. PLoS ONE.

[35] Costa E.C., Gaspar V.M., Coutinho P., Correia I.J. (2014). Optimization of liquid overlay technique to formulate heterogenic 3D co-cultures models. Biotechnol. Bioeng..

[36] Rasband W.S. Image J. http://rsbweb.nih.gov/ij/.

[37] Klaka P., Grüdl S., Banowski B., Giesen M., Sättler A., Proksch P., Welss T., Förster T. (2017). A novel organotypic 3D sweat gland model with physiological functionality. PLoS ONE.

[38] Yu T., Qi Y., Wang J., Feng W., Xu J., Zhu J., Yao Y., Gong H., Luo Q., Zhu D. (2016). Rapid and prodium iodide-compatible optical clearing method for brain tissue based on sugar/sugar-alcohol. J. Biomed. Opt..

[39] Ke M.-T., Fujimoto S., Imai T. (2013). SeeDB: A simple and morphology-preserving optical clearing agent for neuronal circuit reconstruction. Nat. Neurosci..

[40] Decroix L., Van Muylder V., Desender L., Sampaolesi M., Thorrez L. (2015). Tissue clearing for confocal imaging of native and bio-artificial skeletal muscle. Biotech. Histochem..

[41] Chubinskiy-Nadezhdin V.I., Negulyaev Y.A., Morachevskaya E.A. (2017). Simvastatin induced actin cytoskeleton disassembly in normal and transformed fibroblasts without affecting lipid raft integrity. Cell Biol. Int..

[42] Lipskaia L., Hadri L., Prince P.L., Esposito B., Atassi F., Liang L., Glorian M., Limon I., Lompre A.M., Lehoux S. (2013). SERCA2a gene transfer prevents intimal proliferation in an organ culture of human internal mammary artery. Gene Ther..

[43] Tainaka K., Kuno A., Kubota S.I., Murakami T., Ueda H.R. (2016). Chemical principles in tissue clearing and staining protocols for whole-body cell profiling. Annu. Rev. Cell Dev. Biol..

[44] Lee E., Kim H.J., Sun W. (2016). See-Through technology for biological tissue: 3-Dimensional visualization of macromolecules. Int. Neurourol. J..

[45] Fuchs J., Dell’Atti D., Buhot A., Calemczuk R., Mascini M., Livache T. (2010). Effects of formamide on the thermal stability of DNA duplexes on biochips. Anal. Biochem..

[46] Olive P.L., Banath J.P., Fjell C.D. (1994). DNA strand breakage and DNA structure influence staining with propidium iodide using the alkaline comet assay. Cytometry.

[47] Lasagni A., Yuan D., Shao P., Das S. Rapid fabrication of biocompatible hydrogels microdevices using laser interference lithography. Proceedings of the Bioengineered and Bioinspired Systems IV.

[48] Jayaramudu T., Raghavendra G.M., Varaprasad K., Reddy G.V.S., Reddy A.B., Sudhakar K., Sadiku E.R. (2016). Preparation and characterization of poly(ethylene glycol) stabilized nano silver particles by a mechanochemical assisted ball mill process. J. Appl. Polym. Sci..

[49] Nguyen H.N., Ha P.T., Nguyen A.S., Nguyen D.T., Do H.D., Thi Q.N., Thi M.N.H. (2016). Curcumin as fluorescent probe for directly monitoring in vitro uptake of curcumin combined paclitaxel loaded PLA-TPGS nanoparticles. Adv. Nat. Sci. Nanosci. Nanotechnol..

[50] Dias D.R., Moreira A.F., Correia I.J. (2016). The effect of the shape of gold core–mesoporous silica shell nanoparticles on the cellular behavior and tumor spheroid penetration. J. Mater. Chem..

[51] Alves C.G., de Melo-Diogo D., Lima-Sousa R., Costa E.C., Correia I.J. (2019). Hyaluronic acid functionalized nanoparticles loaded with IR780 and DOX for cancer chemo-photothermal therapy. Eur. J. Pharm. Biopharm..

[52] De Sampaio P.C., Auslaender D., Krubasik D., Failla A.V., Skepper J.N., Murphy G., English W.R. (2012). A heterogeneous in vitro three dimensional model of tumour-stroma interactions regulating sprouting angiogenesis. PLoS ONE.

[53] Wei B., Han X.-Y., Qi C.-L., Zhang S., Zheng Z.-H., Huang Y., Chen T.-F., Wei H.-B. (2012). Coaction of spheroid-derived stem-like cells and endothelial progenitor cells promotes development of colon cancer. PLoS ONE.

[54] Barros A.S., Costa E.C., Nunes A.S., de Melo-Diogo D., Correia I.J. (2018). Comparative study of the therapeutic effect of Doxorubicin and Resveratrol combination on 2D and 3D (spheroids) cell culture models. Int. J. Pharm..

